# Association between HLA-A gene polymorphism and early-onset preeclampsia in Chinese pregnant women early-onset

**DOI:** 10.1186/s12884-020-03340-w

**Published:** 2020-10-30

**Authors:** Yuanyuan Zheng, Cui Ma, Xiaowei Liu, Shaowen Wu, Weiyuan Zhang, Shenglong Zhao

**Affiliations:** Department of Obsterics, Beijing Obstetrics and Gynecology Hospital, Capital Medical University, No.251 Yaojiayuan Road, Chaoyang District 100026 Beijing, China

**Keywords:** Human leukocyte antigen, Preeclampsia, HLA-A polymorphism

## Abstract

**Background:**

Preeclampsia is an idiopathic disease during pregnancy. This study explores the correlation between HLA-A polymorphism and the onset of preeclampsia.

**Methods:**

The Illumina HiSeq2500 sequencing platform was used to genotyping HLA-A allele in venous blood DNA of 50 preeclampsia pregnant women and 48 normal pregnant women and umbilical cord blood DNA of their children of Han nationality in China. The frequencies and distributions of alleles and genotypes among the mothers and their children were compared between the two groups. The differences of frequencies and distributions of genotypes were compared between the two groups according to the mothers’ genotype compatibility.

**Results:**

Twenty HLA-A alleles were detected in preeclampsia pregnant women and normal pregnant women; 21 HLA-A alleles were found in preeclampsia group fetuses and 22 HLA-A alleles in control group fetuses. There was no statistical difference in the HLA-A genes’ frequency between the two groups of pregnant women and their fetuses. When the sharing antigen was 1, the number of maternal-fetal pairs in the preeclampsia group was more than that in the control group; the difference was statistically significant (*P* < 0.05). The frequency of neither mother nor fetus carrying the HLA-A * 24: 02 gene in the preeclampsia group was significantly lower than that in the control group (*P *< 0.05). HLA-A gene homozygosity in fetuses of early-onset preeclampsia group was substantially higher than that of the control group (*P* = 0.0148); there is no significant difference in pregnant women’s genes homozygosity between early-onset preeclampsia group and the control group.

**Conclusions:**

HLA-A * 24: 02 may be a susceptibility gene for early preeclampsia.

## Background

Preeclampsia is an idiopathic disease during pregnancy. The clinical manifestations of preeclampsia are hypertension and/or proteinuria after 20 weeks of pregnancy, accompanied by systemic organ function damage and placenta function damage, which seriously endangers mothers and children [1]. It is the leading cause of maternal death and an essential reason for increasing perinatal incidence and mortality. The prevalence of preeclampsia is approximately 1.5–4.6% in the whole world [2]. According to the difference in onset gestational age, pathogenesis, and pathologic characteristics, the preeclampsia can be divided into early-onset preeclampsia and late onset preeclampsia [3]. Early-onset preeclampsia is characterized by early-onset, rapid disease progression, numerous maternal and infant complications, and high maternal and child mortality. The pathogenesis of early-onset preeclampsia has not been cleared yet, the characteristic pathology of early-onset preeclampsia turns into uterine spiral artery remodeling disorder, shallow placenta implantation, and the arterial pathology of the placental bed in patients became similar to acute transplant rejection, which are summarized in placental disease [4].

Human leukocyte antigen (HLA) plays a leading role baby’s in the immune function and participates in the rejection of transplantation. HLA-A belongs to the typical HLA class I-a antigen[4]. It recognizes and presents endogenous antigens, triggers allogeneic rejection, and has a high degree of genetic polymorphism. This study explores the relationship between HLA-A polymorphism and the early-onset preeclampsia, and lays the foundation for further elucidating the pathogenesis of early-onset preeclampsia.

## Methods

### Research subjects

A total of 98 pregnant women delivered in Beijing Maternity Hospital from October 2017 to October 2019 were included in our present study. 50 cases of early-onset preeclampsia women with gestational age ≤ 34 weeks were selected as the experimental group, and 48 cases of normal pregnant women who delivered at the same time were selected as the control group. The average age of preeclampsia pregnant women was 30.60 ± 5.76 years old, and the average pregnancy was 31.95 ± 2.22 weeks. The average age of pregnant women in the normal group was 30.28 ± 4.19 years old and 30.23 ± 2.10 weeks of gestation. There was no significant difference in age, gestational weeks and parity between the two groups. The exclusion criteria: age < 18 years; nationality except for Han; autoimmune disorders (including systemic lupus erythematosus, diabetes mellitus type I, rheumatoid arthritis, antiphospholipid syndrome, hyperthyroidism); the history of habitual abortion (≥ 3 times); previous chronic hypertension; kidney disease; multiple pregnancies; severe fetal malformation; intrauterine fetal death.

According to the Guidelines for the Diagnosis and Treatment of Hypertensive Diseases During Pregnancy (2015), the diagnostic criteria for preeclampsia were blood pressure rises after 20 weeks of pregnancy, systolic blood pressure ≥ 140 mmHg and / or diastolic blood pressure ≥ 90 mmHg, accompanied by any of the following: proteinuria ≥ 0.3 g/24 h, or proteinuria / creatinine ratio ≥ 0.3, or random proteinuria ≥ (+); no proteinuria but with the involvement of any of the following organs (heart, lung, liver, kidney and other vital organs) or systems (hematological system, digestive system, nervous system), placenta fetus involvement, etc. All subjects and their families signed informed consent.

## Methods

2 mL of venous blood was drawn from pregnant women before delivery and loaded into EDTA vacuum blood collection tubes. 2 mLof fetal umbilical cord blood was drawn into EDTA vacuum tubes within 5 minutes after birth. The specimens were stored in refrigerators at -80 °C.

The thawed whole blood and red blood cell lysis buffer was added to a centrifuge tube; the centrifuge tube contents were mixed with a vortex mixer. After centrifugation for 10 minutes, the supernatant was discarded. The nuclear cell lysate, protease K, SDS were added into precipitate and mixed well, then the centrifuge tube was placed in a thermostatic mixer, and the mixture was lysed for 60 min. Cooling after lysing, phenol / chloroform / isoamyl alcohol was added and centrifugated for another 10 minutes. The supernatant was transferred into a new 1.5 mL centrifuge tube with precooled isopropanol and sodium acetate, put it into the refrigerator at − 20 ℃ for overnight precipitation. One the next day, after centrifugation for 10 minutes, discard the supernatant, added ethanol. The precipitate was rinsed with a pipette, then it was centrifuged for 5 minutes, and the supernatant was discarded. Centrifuged briefly again, aspirated any residual liquid, and dried. The precipitate was soluble in the proper amount of TE.

DNA quantification was performed by fluorescence quantification and agarose gel electrophoresis. A qualified DNA sample was interrupted to a 500 bp main peak, with the “A” base at the 3 ‘end, and a library adapter at both ends. Linear amplification was used to create a hybrid library captured and enriched with the MHC capture chip. The Illumina HiSeq2500 sequencing platform was used, and the effective average sequencing depth was not less than 80X.

### Statistics analysis

Plink statistical software was used to analyze the gene frequency. The allele and genotype frequencies of each test site were calculated, and the Hardy-Weinberg equilibrium was verified [5]. Pearson’s chi-squared test or Chi-square test of continuity correction or Fisher exact probability method was used to calculating the gene frequency difference between the two groups. A *P* < 0.05 represents significant statistical difference.

## Results

### Distribution of HLA-A alleles in early-onset preeclampsia pregnant women and normal pregnant women

20 HLA-A alleles were detected in 50 cases of early-onset preeclampsia pregnant women in the experimental group. The alleles accounting for more than 10% were HLA-A * 24: 02 (20%), -A * 02: 01 (15%), -A * 11: 01 (12%), dominant alleles were not found. 20 HLA-A alleles were detected in 48 pregnant women in the control group. The alleles accounting for more than 10% were HLA-A * 02: 01 (15%), -A * 11: 01 (14%), -A * 02: 06 (10%), dominant alleles were not found. The proportion of HLA-A * 24: 02 (20%) genes in early-onset preeclampsia pregnant women was significantly higher than that of normal pregnant women (9.38%), but the difference didn’t have statistically significant. See Table 1 for details.

**Table 1:** Comparison of HLA-A allele frequency distribution among mothers in preeclampsia group and control group

Gene	Alleles	Control Group	Preeclampsia Group
A	A*01:01	5(5.21%)	3(3.00%)
	A*01:22	0(0.00%)	2(2.00%)
	A*02:01	15(15.62%)	15(15.00%)
	A*02:03	1(1.04%)	2(2.00%)
	A*02:05	1(1.04%)	2(2.00%)
	A*02:06	10(10.42%)	9(9.00%)
	A*02:07	8(8.33%)	5(5.00%)
	A*02:10	1(1.04%)	1(1.00%)
	A*02:17	0(0.00%)	0(0.00%)
	A*02:48	1(1.04%)	0(0.00%)
	A*03:01	3(3.12%)	6(6.00%)
	A*03:02	1(1.04%)	1(1.00%)
	A*11:01	14(14.58%)	12(12.00%)
	A*11:02	1(1.04%)	0(0.00%)
	A*24:02	9(9.38%)	20(20.00%)
	A*24:03	0(0.00%)	0(0.00%)
	A*24:20	0(0.00%)	0(0.00%)
	A*26:01	4(4.17%)	1(1.00%)
	A*29:01	1(1.04%)	1(1.00%)
	A*30:01	9(9.38%)	6(6.00%)
	A*31:01	5(5.21%)	5(5.00%)
	A*32:01	4(4.17%)	1(1.00%)
	A*33:03	2(2.08%)	6(6.00%)
	A*34:01	0(0.00%)	1(1.00%)
	A*66:01	0(0.00%)	0(0.00%)
	A*68:01	1(1.04%)	1(1.00%)
	A(Total)	96(100.00%)	100(100.00%)

### Distribution of HLA-A alleles in early-onset preeclampsia group fetuses and normal group fetuses

21 HLA-A alleles were detected in 50 cases of early-onset preeclampsia group fetuses. The alleles accounting for more than 10% were HLA-A * 02: 01 (20%), -A * 24: 02 (19%), -A * 11: 01 (14%), dominant alleles were not found. 22 HLA-A alleles were detected in 48 fetuses in the control group. The alleles accounting for more than 10% were HLA-A * 11: 01 (17.71%), -A * 02: 01 (15.62%), -A * 24: 02 (12.5%), dominant alleles were not found. The proportion of HLA-A * 24: 02 (19%) genes in early-onset preeclampsia group fetus were significantly higher than that in the control group fetus (12.5%), but the difference didn’t have statistically significant. See Table 2 for details.

**Table 2:** Comparison of HLA-A allele frequency distribution in fetuses in preeclampsia group and control group

Gene	Alleles	Control Group	Preeclampsia Group
A	A*01:01	3(3.12%)	3(3.00%)
	A*01:22N	2(2.08%)	1(1.00%)
	A*02:01	15(15.62%)	20(20.00%)
	A*02:03	0(0.00%)	0(0.00%)
	A*02:05	2(2.08%)	2(2.00%)
	A*02:06	8(8.33%)	5(5.00%)
	A*02:07	5(5.21%)	6(6.00%)
	A*02:10	1(1.04%)	1(1.00%)
	A*02:17	1(1.04%)	0(0.00%)
	A*02:48	1(1.04%)	0(0.00%)
	A*03:01	3(3.12%)	4(4.00%)
	A*03:02	1(1.04%)	1(1.00%)
	A*11:01	17(17.71%)	14(14.00%)
	A*11:02	2(2.08%)	1(1.00%)
	A*24:02	12(12.50%)	19(19.00%)
	A*24:03	1(1.04%)	0(0.00%)
	A*24:20	0(0.00%)	1(1.00%)
	A*26:01	2(2.08%)	2(2.00%)
	A*29:01	1(1.04%)	1(1.00%)
	A*30:01	8(8.33%)	8(8.00%)
	A*31:01	3(3.12%)	5(5.00%)
	A*32:01	3(3.12%)	2(2.00%)
	A*33:03	4(4.17%)	2(2.00%)
	A*34:01	0(0.00%)	0(0.00%)
	A*66:01	0(0.00%)	1(1.00%)
	A*68:01	1(1.04%)	1(1.00%)
	A(Total)	96(100.00%)	100(100.00%)

### Comparison of HLA-A antigen sharing frequency between mother and fetus in early-onset preeclampsia and control group

The HLA gene is a multiple alleles; there are two antigenic phenotypes in each HLA locus on a homologous chromosome pair. One-half of the fetal genes are from the mother, and the other half is from the father, so there must be 1 or 2 identical HLA-A phenotypes between the mother and the fetus. If there is one identical antigens between the mother and fetus, the number of shared antigens is recorded as 1. If there are two identical antigens between the mother and fetus, the number of shared antigens is recorded as 2.

There were five pairs of mothers and fetuses having two shared antigens in the preeclampsia group, accounting for 10% (5/50). There were 44 pairs of mothers and fetuses having one shared antigen in the preeclampsia group, accounting for 88% (44/50). There were 13 pairs of mothers and fetuses having 2 shared antigens in the preeclampsia group, accounting for 27.08% (13/48). There were 35 pairs of mothers and fetuses having 1 shared antigen in the preeclampsia group, accounting for 72.92% (35/48). The difference was statistically significant (*P* < 0.05). See Table 3 for details.

**Table 3:** HLA-A antigen sharing in preeclampsia group and control group

HLA Gene	Antigen Sharing	Preeclampsia Group	Control Group	OR (CI)	*P*^a^
HLA-A	1	44(88%)	35(72.92%)	3.27(1.06,10.05)	0.0391
	2	5(10%)	13(27.08%)	0.31(0.10,0.94)	0.0391

Note: ^a^Fisher exact test was used.

### Analysis of the compatibility frequency of HLA-A alleles between mothers and fetuses in early-onset preeclampsia group and control group

The higher allele frequency (> 10%) (HLA-A * 11: 01, -A * 02: 01, -A * 24: 02) in early-onset preeclampsia group and control group were selected to analyze these genes compatibility between mother and child. The results showed that the frequency of neither mother nor fetus carrying the HLA-A * 24: 02 gene in the preeclampsia group was significantly lower than that in the control group (*P *< 0.05). See Table 4 for details.

**Table 4:** Compatibility frequency of some alleles carried by mother and fetus in preeclampsia group and control group

Maternal / Fetal Genes	Maternal genotype / fetal genotype	Preeclampsia Group	Control Group	OR(CI)	*P*-value
A*24:02	--	27	36	0.39(0.17,0.92)	0.0363
A*24:02	+-	7	1	7.65(0.90,64.75)	0.0597
A*24:02	++	11	6	1.97(0.67,5.85)	0.288
A*24:02	-+	5	5	0.96(0.26,3.53)	1.00
A*11:01	++	6	8	0.68(0.22,2.14)	0.573
A*11:01	--	31	28	1.17(0.52,2.62)	0.837
A*11:01	+-	5	4	1.22(0.31,4.85)	1.00
A*11:01	-+	8	8	0.95(0.33,2.78)	1.00
A*02:01	+-	2	5	0.36(0.07,1.94)	0.264
A*02:01	--	34	28	1.52(0.66,3.47)	0.403
A*02:01	++	9	10	0.83(0.31,2.27)	0.801
A*02:01	-+	5	5	0.96(0.26,3.53)	1.00

### Comparison of HLA-A allele homozygosity between mother and fetus in early-onset preeclampsia group and control group

When two alleles at the same locus of a homologous chromosome are the same, they are called homozygous. When two alleles at the same locus of a homologous chromosome are not the same, they are heterozygous. The results showed that the HLA-A gene homozygosity in fetuses of the early-onset preeclampsia group was significantly higher than that of the control group (*P *= 0.0148). There is no significant difference in pregnant women’s gene homozygosity between the early-onset preeclampsia and the control groups. See Table 5 for details.

**Table 5:** HLA homozygous / heterozygous distribution of mother and fetus

Gene	Neonate			Pregnant Woman		
	Control Group	Preeclampsia Group	*P *	Control Group	Preeclampsia Group	*P *
HLA-A	48(100.00%)	50(100.00%)		48(100.00%)	50(100.00%)	
Homozygous	2(4.17%)	11(22.00%)	0.0148	4(8.33%)	7(14.00%)	0.525
Heterozygous	46(95.83%)	39(78.00%)		44(91.67%)	43(86.00%)	

## Discussion

The children carry half of the HLA genes from their mothers and a half from their fathers [6]. During pregnancy, the fetus carrying the paternal HLA gene will be regarded as a homograft. Under normal circumstances, the pregnancy can continue smoothly because of the immune tolerance between mother and fetus. Once the immune tolerance balance is broken, immune abnormalities occur at the maternal-fetal interface, which leads to the reduced invasive capacity of placental trophoblasts, recast dysfunction of the spiral uterine artery, damage to the vascular endothelium, the release of inflammatory factors [7]. These changes were similar to transplant rejection reactions. The fetus will be treated as allogeneic. Simultaneously, the mother has hypertension, proteinuria, impaired organ function, fetus’s growth retardation, and low body weight [8].

Aa classic HLA-I antigen, HLA-A is distributed on the surface of almost all nucleated cells in the body. Its primary function is to present endogenous antigens to CD8 + T lymphocytes to participate in specific immune responses and the genetic regulation of immune responses. Many scholars have studied the HLA-A and preeclampsia, but the results were not the same [9]. Biggar [10] found that HLA-A gene sharing and distribution frequency between mother and fetus were not significantly different in the preeclampsia group than normal pregnant women. However, Triche et al. [11] analyzed the relationship between pregnant women in the preeclampsia group and the semen exposure time, and found that the effect of HLA-A gene sharing between mothers and fetuses in preeclampsia pregnant women in the low semen exposure group increased significantly (OR = 6.27, 95% CI is 1.04, 37.99). Emmery et al. [12] found no significant correlation in HLA-A gene sharing between preeclampsia maternal and fetal after correcting the body mass index and smoking factors. however, the homozygous rate of HLA-A gene loci in preeclampsia pregnant women was the corresponding rise.

There are several findings in our research. (1) There was no significant difference in the distribution of HLA-A gene frequency between the preeclampsia and control groups. The results were similar to those of Zhang et al. In their study on the association between preeclampsia HLA-A gene polymorphism and preeclampsia, there was no significant difference in HLA-A allele between preeclampsia group and control group (P (c) > 0.05) [13].But we still observed that the frequency of HLA-A * 24: 02 gene in pregnant women and fetuses in the preeclampsia group is about 10% higher than that in the control group. Moreover, the number of neither mother nor fetus not carrying this gene in the healthy pregnancy group was higher than that in the early-onset preeclampsia group. Whether HLA-A * 24: 02 is a preeclampsia susceptibility gene needs to expand the sample for further research. (2) In the HLA-A gene sharing between mother and fetus, the gene sharing between mother and fetus in the control group (OR = 0.31,95% CI = 0.10,0.94) was more than that in early-onset preeclampsia group, which suggests that the HLA-A gene sharing between mother and fetus is conducive to the maternal and fetal immune tolerance of pregnancy and protect the fetus from maternal rejection. Robert J et al. analyzed 201 pairs of maternal-infant HLA-A and found that maladaptation mediated by adaptive immunity between mother and infant was not based on maternal development of preeclampsia/eclampsia [14]. This view is also found in a questionnaire survey on the relationship between donors in donor pregnancy [13]. Compared with family-related donors, when donors were not related to recipients, hypertension incidence during pregnancy has increased. It is also suggested that HLA histocompatibility between the mother and fetus was beneficial to a healthy pregnancy. (3) Fetal HLA-A homozygosity was associated with preeclampsia. The results are consistent with those of Redman et al [15]. The possible reason is that the mother will have an immune tolerance response after contacting the HLA antigen of the sexual partner’s semen, but the HLA homozygote of the fetus will reduce the heterogeneity of the antigen between couples, reduce the mother’s immune tolerance formation and weaken immune protection, thereby inducing the occurrence of preeclampsia. (4) The father plays an important role in the onset of preeclampsia. Pregnancy can be regarded as a successful semi-allograft. As a half-barrel allograft, the fetus evades the immune attack of the mother, which plays a very important role in the immune recognition of HLA antigens expressed by fetal cells from the paternal side [16]. Some specific gene compatibility patterns between couples may be related to the onset of preeclampsia. Paternal genes may result in immune disorders between mother and fetus, immune intolerance, and rejection of the fetus, which by insufficient blocking antibodies caused by antigen sharing between couples, and too few protective antibodies produced by the mother; it may also be through susceptible genes, and immunological dysfunction caused by gene linkage imbalance leads to preeclampsia. The specific mechanism needs to explored from expanding the sample size and the whole family in the future. Due to the limitation of the number of cases in this study, the relationship between paternal HLA-A allele and the sharing of HLA-A gene between couples and preeclampsia needs to be expanded and further study.

## Conclusions

In conclusion, HLA-A * 24: 02 may be a susceptibility gene for early preeclampsia. This gene may have a greater impact on the occurrence and development of early preeclampsia. Therefore, more detailed research of this gene are of great significance for the prevention of early preeclampsia.

## Abbreviation

HLA: Human leukocyte antigen
